# Supplementary material for: Research on the management of the system construction of National parks with China characteristics: Evidence from policy texts
Source: PLoS One. 2026 Mar 2;21(3):e0340874. doi: 10.1371/journal.pone.0340874 (PMC12952615; doi:10.1371/journal.pone.0340874)
Supplement: S1 Appendix — This table presents the hierarchical coding framework developed for the systematic analysis of national park policy documents. The framework is structured around seven primary variables (X1-X7) and 49 secondary indicators. Each primary variable is subdivided into secondary variables, which are further defined through specific, clearly delineated fundamental categories. (DOCX) [file pone.0340874.s001.docx]

**Appendix 1 Variable settings in the quantitative evaluation system for national park policies in China.**

| **Principal variables** | **No. of Secondary variables** | **Secondary variables** | **Main Basic Category** |
| --- | --- | --- | --- |
| Policy basis  X_1_ | X_1-1_ | Guiding ideology | Ecological civilization ideology ; New development concept; Socialist ideology |
|  | X_1-2_ | Realistic problems | Multiple management; Fragmentation issues |
|  | X_1-3_ | Basic principle | Principles of plan protection and utilization control; Authenticity; Completeness |
|  | X_1-4_ | National strategy | Chinese characteristics; Beautiful China |
| Policy objectives X_2_ | X_2-1_ | Ecological protection and restoration | Ecological protection; Ecological restoration |
|  | X_2-2_ | Realizing the value of ecological products | Universal sharing; Ecological value |
|  | X_2-3_ | Improvement of people's livelihood and well-being | Income increase; Employment growth； Domestic sewage treatment; Medical assistance; Distance learning |
|  | X_2-4_ | Showcasing the responsibility of great powers | International first-class; Leading globally |
|  | X_2-5_ | Local knowledge inheritance | Cultural relic protection; Intangible cultural heritage protection |
|  | X_2-6_ | Environmental justice | Harmonious coexistence between humans and nature; Community of life between humans and nature |
| Policy measures  X_3_ | X_3-1_ | Pilot demonstration | System pilot; Demonstration Village； Outstanding contributions; |
|  | X_3-2_ | Planning and construction | Overall planning; Park layout; Ecological corridor |
|  | X_3-3_ | Technology support | Technology; Drones; Informatization; |
|  | X_3-4_ | Evaluation monitoring | Ecological monitoring; Dynamic supervision |
|  | X_3-5_ | Effectiveness evaluation | Performance evaluation; Ecological monitoring; Dynamic supervision |
|  | X_3-6_ | Industrial transformation and upgrading | Franchise operation; Ecotourism; Green development; Transformation and upgrading |
|  | X_3-7_ | Complete facilities | Facilities; Equipment; Platform |
|  | X_3-8_ | Actively cooperate | Direct exercise; Implement and implement; Refine task division of labor |
|  | X_3-9_ | Improving quality and efficiency | Unified, standardized, and efficient; High quality; Steadily advancing; |
|  | X_3-10_ | Natural disaster prevention and control | Disaster prevention and reduction; Natural disasters; Biological control; |
|  | X_3-11_ | Green consumption | Reasonable utilization; Green procurement; Code of conduct |
|  | X_3-12_ | Ecological relocation | Ecological relocation; Ecological immigration; Migrant residents |
|  | X_3-13_ | Ecological compensation | Horizontal compensation; Financial compensation; Subsidy standards |
|  | X_3-14_ | Tailor measures to local conditions | Features; Suitable; Gradual transition |
| Policy receptors X_4_ | X_4-1_ | Governments | Local governments; The central government |
|  | X_4-2_ | NGO | Non governmental organizations; International organization |
|  | X_4-3_ | Enterprise | Cultural enterprises; Forest enterprises |
|  | X_4-4_ | Community residents | Indigenous peoples; Local residents |
|  | X_4-5_ | Research institutes | Experts and scholars; Higher education institutions; Academic exchange; |
|  | X_4-6_ | The public | Volunteers; All sectors of society; Social organization |
| Policy areas  X_5_ | X_5-1_ | Political field | National park system; Public utilities; National interests |
|  | X_5-2_ | Economic field | Industrial operation; Fiscal revenue; Financial financing |
|  | X_5-3_ | Social field | Human society ;Future generations |
|  | X_5-4_ | Culture field | Cultural heritage; Intangible cultural heritage |
|  | X_5-5_ | Ecological field | Wildlife; Wild plants |
| Policy functions X_6_ | X_6-1_ | Prediction | Three years; By 2035 |
|  | X_6-2_ | Regulation | Supervision; Monitoring; Audit |
|  | X_6-3_ | Suggestion | Measures; Method; Opinions; |
|  | X_6-4_ | Description | Total area; Population size; Small scale |
|  | X_6-5_ | Diagnosis | Correct; Distinguish; Explore |
|  | X_6-6_ | Guidance | Support; Promote |
|  | X_6-7_ | Identification | Inclusion; Boundary; Identification; Logo; |
|  | X_6-8_ | Specification | Standardization; Prohibited; Control |
| Policy guarantees X_7_ | X_7-1_ | Organizational leadership | Management organization; Establishing institutions; Competent department |
|  | X_7-2_ | Institutional guarantees | Reporting system; Evaluation system; Rights protection mechanism |
|  | X_7-3_ | Personnel training | Talent cultivation; Team building; Technical training; Professional personnel |
|  | X_7-4_ | Financial support | Financial input; Social capital |
|  | X_7-5_ | Laws and regulations | Enforcement in accordance with the law; Strict law enforcement; Comprehensive law enforcement; Violation of laws and regulations |
|  | X_7-6_ | Publicity and guidance | Propaganda column; Television communication; Video imaging |
