# Supplementary material for: Research on the management of the system construction of National parks with China characteristics: Evidence from policy texts
Source: PLoS One. 2026 Mar 2;21(3):e0340874. doi: 10.1371/journal.pone.0340874 (PMC12952615; doi:10.1371/journal.pone.0340874)
Supplement: S2 Appendix — This conceptual model visually maps the hypothesized pathways of influence among the seven core policy dimensions identified in the analytical framework (S1 Appendix). The diagram depicts a sequential and interactive logic: Policy Basis stimulates the formulation of Policy Objectives. These objectives are to be achieved through specific Policy Measures, which in turn ensure the implementation of Policy Guarantees. This combined policy apparatus acts within defined Policy Areas and on key Policy Receptors. The ultimate outcomes of this entire process are the realization of various Policy Functions. (PDF) [file pone.0340874.s002.pdf]

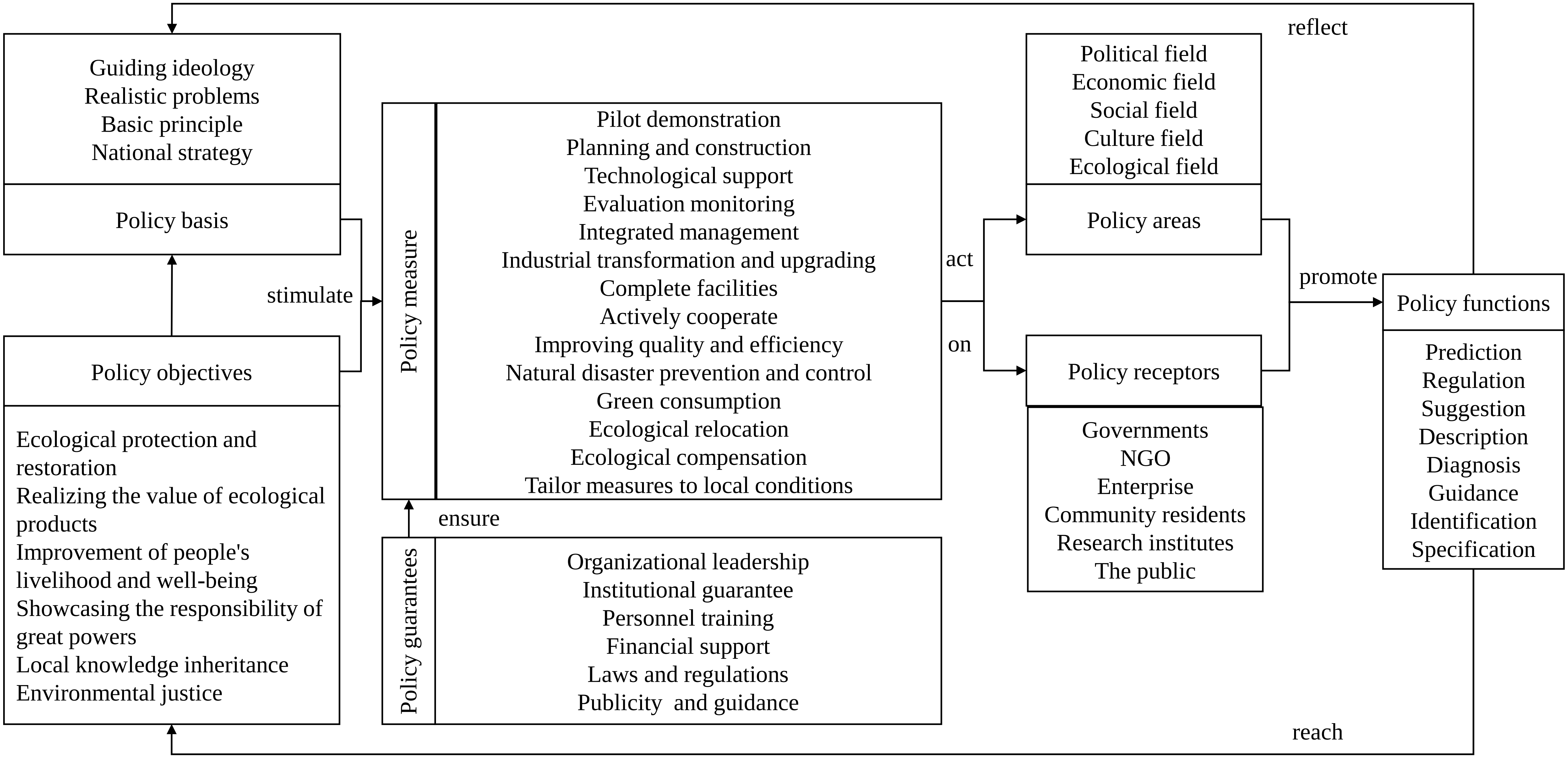

Appendix 2 Study constitute the proposed framework for quantitative analysis of China’s national park policy texts.
