# Supplementary material for: Research on the management of the system construction of National parks with China characteristics: Evidence from policy texts
Source: PLoS One. 2026 Mar 2;21(3):e0340874. doi: 10.1371/journal.pone.0340874 (PMC12952615; doi:10.1371/journal.pone.0340874)
Supplement: S3 Appendix — This table presents the average scores for 49 secondary policy variables, calculated separately for aggregated central-level and local-level policy documents. The variables span all seven principal dimensions of the analytical framework. For each variable, the table lists its average score at the central level, its average score at the local level, and the standard deviation between these two values. The standard deviation quantifies the degree of divergence in emphasis on each specific policy element between the two administrative tiers. (DOCX) [file pone.0340874.s003.docx]

**Appendix 3 Average scores and standard deviations of secondary variables between two levels.**

| **Secondary variables** | **Central level average scores** | **Local level average scores** | **Standard deviation** | **Secondary variables** | **Central level average scores** | **Local level average scores** | **Standard deviation** |
| --- | --- | --- | --- | --- | --- | --- | --- |
| X_1-1_ | 1.26 | 2.04 | 0.55 | X_4-1_ | 0.89 | 5.43 | 3.21 |
| X_1-2_ | 5.13 | 5.03 | 0.08 | X_4-2_ | 1.4 | 2.94 | 1.09 |
| X_1-3_ | 1.59 | 1.99 | 0.29 | X_4-3_ | 1.75 | 3 | 0.88 |
| X_1-4_ | 2.46 | 3.24 | 0.55 | X_4-4_ | 2.92 | 14.39 | 8.11 |
| X_2-1_ | 5.53 | 24.5 | 13.41 | X_4-5_ | 0.47 | 2.44 | 1.39 |
| X_2-2_ | 2.09 | 3.18 | 0.77 | X_4-6_ | 2.27 | 2.92 | 0.46 |
| X_2-3_ | 2.43 | 14.29 | 8.39 | X_5-1_ | 0.82 | 2.86 | 1.44 |
| X_2-4_ | 0.6 | 3.38 | 1.96 | X_5-2_ | 2.83 | 10.38 | 5.34 |
| X_2-5_ | 0.77 | 1.68 | 0.64 | X_5-3_ | 1.13 | 2.66 | 1.08 |
| X_2-6_ | 1.12 | 3.24 | 1.5 | X_5-4_ | 0.17 | 1.17 | 0.71 |
| X_3-1_ | 2.79 | 10.22 | 5.26 | X_5-5_ | 33.68 | 177.22 | 101.5 |
| X_3-2_ | 27.7 | 90.94 | 44.72 | X_6-1_ | 1.13 | 3.71 | 1.82 |
| X_3-3_ | 2.47 | 6.34 | 2.74 | X_6-2_ | 1.37 | 3.86 | 1.76 |
| X_3-4_ | 10.65 | 13.55 | 2.05 | X_6-3_ | 1.3 | 1.95 | 0.46 |
| X_3-5_ | 62.24 | 146.28 | 59.42 | X_6-4_ | 4.43 | 14.47 | 7.1 |
| X_3-6_ | 14.67 | 52.57 | 26.79 | X_6-5_ | 2.84 | 2.78 | 0.04 |
| X_3-7_ | 3.34 | 13.61 | 7.27 | X_6-6_ | 0.78 | 1.8 | 0.72 |
| X_3-8_ | 0.94 | 2.8 | 1.32 | X_6-7_ | 3.17 | 6.3 | 2.21 |
| X_3-9_ | 7.29 | 25.6 | 12.95 | X_6-8_ | 8.55 | 25.43 | 11.94 |
| X_3-10_ | 0.3 | 1.87 | 1.11 | X_7-1_ | 13.73 | 28.6 | 10.51 |
| X_3-11_ | 1.76 | 6.27 | 3.19 | X_7-2_ | 2.99 | 9.43 | 4.55 |
| X_3-12_ | 0.97 | 3.64 | 1.88 | X_7-3_ | 0.57 | 2.33 | 1.24 |
| X_3-13_ | 1.79 | 4.51 | 1.93 | X_7-4_ | 1.51 | 1.45 | 0.04 |
| X_3-14_ | 2.03 | 5.47 | 2.43 | X_7-5_ | 1.36 | 3.59 | 1.58 |
| — | | | | X_7-6_ | 1.62 | 5.71 | 2.89 |
