# Supplementary material for: Research on the management of the system construction of National parks with China characteristics: Evidence from policy texts
Source: PLoS One. 2026 Mar 2;21(3):e0340874. doi: 10.1371/journal.pone.0340874 (PMC12952615; doi:10.1371/journal.pone.0340874)
Supplement: S4 Appendix — This table provides the reference codes, full names, and release periods for the key national-level policy documents on National Park planning that form the core of the policy analysis. The listed master plans, including those for the Northeast Tiger and Leopard, Sanjiangyuan, Hainan Tropical Rainforest, Wuyishan, and Giant Panda National Parks, represent recent, long-term strategic frameworks and serve as primary sources for the subsequent textual and content analysis. (DOCX) [file pone.0340874.s004.docx]

**Appendix 4 Samples of master plans for China's first five national parks.**

| **No.** | **Name of policy** | **Release time** |
| --- | --- | --- |
| P7 | Northeast Tiger and Leopard National Park Master Plan (2022-2030) | 2022 |
| P9 | Sanjiangyuan National Park Master Plan (2023-2030) | 2023 |
| P19 | Hainan Tropical Rainforest National Park Master Plan (2022-2030) | 2022 |
| P23 | Wuyishan National Park Master Plan (2023-2030) | 2023 |
| P26 | General Regulations of the Giant Panda National Park (2023-2030) | 2023 |
