# Supplementary material for: Research on the management of the system construction of National parks with China characteristics: Evidence from policy texts
Source: PLoS One. 2026 Mar 2;21(3):e0340874. doi: 10.1371/journal.pone.0340874 (PMC12952615; doi:10.1371/journal.pone.0340874)
Supplement: S5 Appendix — This table reports the quantified metrics (e.g., frequency counts or standardized scores) for 49 secondary variables extracted from the content analysis of five key policy documents (P7, P9, P19, P23, P26). The final column provides the standard deviation for each variable, reflecting the variation in its prominence across the different plans. (DOCX) [file pone.0340874.s005.docx]

**Appendix 5 Secondary variable scores and standard deviations for the five national park policy texts.**

| **Secondary variables** | **P7** | **P9** | **P19** | **P23** | **P26** | **Standard Deviation** |
| --- | --- | --- | --- | --- | --- | --- |
| X_1-1_ | 0.16 | 3.42 | 2.11 | 3.74 | 3.09 | 1.44 |
| X_1-2_ | 2.57 | 0.00 | 1.28 | 10.27 | 8.98 | 4.68 |
| X_1-3_ | 1.03 | 2.40 | 1.72 | 3.78 | 2.06 | 1.02 |
| X_1-4_ | 0.00 | 3.28 | 1.64 | 6.02 | 6.02 | 2.66 |
| X_2-1_ | 10.19 | 24.00 | 46.37 | 86.81 | 44.23 | 29.01 |
| X_2-2_ | 1.37 | 2.90 | 4.67 | 4.34 | 4.83 | 1.47 |
| X_2-3_ | 4.51 | 12.66 | 20.12 | 36.25 | 20.46 | 11.73 |
| X_2-4_ | 0.51 | 1.88 | 3.08 | 3.93 | 6.33 | 2.19 |
| X_2-5_ | 0.13 | 0.90 | 1.29 | 8.52 | 1.16 | 3.45 |
| X_2-6_ | 2.45 | 3.27 | 2.04 | 6.95 | 6.54 | 2.32 |
| X_3-1_ | 2.28 | 8.11 | 16.34 | 26.35 | 16.60 | 9.18 |
| X_3-2_ | 42.20 | 65.12 | 187.21 | 229.11 | 153.44 | 79.71 |
| X_3-3_ | 1.39 | 6.30 | 13.74 | 17.15 | 12.48 | 6.30 |
| X_3-4_ | 4.44 | 11.02 | 23.10 | 50.03 | 26.01 | 17.52 |
| X_3-5_ | 71.04 | 126.81 | 234.37 | 372.47 | 205.82 | 115.03 |
| X_3-6_ | 20.66 | 25.97 | 67.00 | 174.73 | 69.07 | 61.93 |
| X_3-7_ | 3.99 | 11.89 | 29.37 | 38.19 | 24.31 | 13.67 |
| X_3-8_ | 1.48 | 2.69 | 4.40 | 8.24 | 4.43 | 2.55 |
| X_3-9_ | 8.02 | 28.83 | 35.12 | 60.91 | 41.62 | 19.24 |
| X_3-10_ | 1.31 | 1.57 | 3.44 | 4.26 | 3.74 | 1.34 |
| X_3-11_ | 0.76 | 4.19 | 9.39 | 14.59 | 9.13 | 5.31 |
| X_3-12_ | 2.13 | 2.13 | 10.37 | 13.29 | 2.66 | 5.32 |
| X_3-13_ | 2.46 | 2.16 | 0.31 | 6.47 | 6.78 | 2.85 |
| X_3-14_ | 1.70 | 4.13 | 9.87 | 13.54 | 9.06 | 4.73 |
| X_4-1_ | 8.30 | 2.26 | 7.76 | 17.32 | 6.77 | 5.49 |
| X_4-2_ | 3.61 | 3.21 | 1.20 | 5.62 | 4.42 | 1.63 |
| X_4-3_ | 1.31 | 0.33 | 2.63 | 10.18 | 2.30 | 3.92 |
| X_4-4_ | 6.15 | 4.61 | 30.20 | 37.75 | 13.98 | 14.78 |
| X_4-5_ | 1.08 | 1.90 | 3.74 | 4.78 | 7.45 | 2.51 |
| X_4-6_ | 1.39 | 2.54 | 3.46 | 9.62 | 4.31 | 3.19 |
| X_5-1_ | 1.13 | 2.64 | 1.70 | 3.77 | 6.59 | 2.16 |
| X_5-2_ | 2.03 | 6.76 | 15.05 | 29.08 | 17.08 | 10.41 |
| X_5-3_ | 2.04 | 2.04 | 3.84 | 7.99 | 3.42 | 2.44 |
| X_5-4_ | 0.32 | 0.68 | 1.66 | 1.72 | 2.04 | 0.74 |
| X_5-5_ | 80.40 | 153.84 | 317.33 | 527.99 | 361.78 | 176.78 |
| X_6-1_ | 0.62 | 1.85 | 9.26 | 2.47 | 3.09 | 3.37 |
| X_6-2_ | 3.21 | 2.01 | 5.49 | 9.63 | 6.82 | 3.01 |
| X_6-3_ | 0.62 | 1.24 | 2.87 | 6.83 | 2.33 | 2.43 |
| X_6-4_ | 2.70 | 12.32 | 26.52 | 52.45 | 24.99 | 18.75 |
| X_6-5_ | 1.89 | 1.26 | 4.89 | 9.62 | 3.78 | 3.31 |
| X_6-6_ | 0.68 | 1.39 | 2.94 | 4.46 | 2.91 | 1.48 |
| X_6-7_ | 3.05 | 4.46 | 9.63 | 18.55 | 9.04 | 6.07 |
| X_6-8_ | 19.60 | 20.00 | 44.60 | 85.20 | 27.40 | 27.56 |
| X_7-1_ | 13.17 | 30.36 | 59.33 | 77.61 | 44.61 | 24.98 |
| X_7-2_ | 5.13 | 12.03 | 12.18 | 20.39 | 13.35 | 5.42 |
| X_7-3_ | 1.01 | 3.04 | 5.07 | 5.47 | 4.16 | 1.79 |
| X_7-4_ | 0.42 | 0.59 | 0.76 | 1.52 | 0.76 | 0.42 |
| X_7-5_ | 8.25 | 3.50 | 4.18 | 4.74 | 3.84 | 1.92 |
| X_7-6_ | 1.48 | 5.04 | 9.96 | 14.26 | 13.03 | 5.41 |
