# Supplementary material for: Research on the management of the system construction of National parks with China characteristics: Evidence from policy texts
Source: PLoS One. 2026 Mar 2;21(3):e0340874. doi: 10.1371/journal.pone.0340874 (PMC12952615; doi:10.1371/journal.pone.0340874)
Supplement: Attachment — Submitted filename: PONE-D-24-46176.docx [file pone.0340874.s007.docx]

Dear Editor,

I am writing to provide my review of the manuscript titled “Research on the Management of the System Construction of National Parks with China Characteristics: Evidence from Policy Texts”. The manuscript examines the development and management of China’s national park system through a quantitative evaluation of 77 policy texts issued between 2013 and 2023. Using a mixed-method approach that combines natural language processing, grounded theory, and statistical analysis, the study compares the central government’s top-level design with local government responses and identifies heterogeneity across the first five national parks. The findings highlight both alignment and divergence in policy objectives and practices, offering insights for refining China’s national park governance and contributing to global discussions on conservation policy.

While the manuscript introduces a technically innovative framework with promising results, I believe that several revisions could improve its clarity, interpretability, and impact. Below, I offer my detailed comments and recommendations:

1. **Abstract:**

- The abstract should include a sentence on the significance of this study for broader international audiences (why non-China readers should care).
- Some sentences in the abstract are long and could be shortened for clarity.

2. **Introduction:**

- The introduction is lengthy and contains excessive descriptive detail (e.g., average park sizes). Consider condensing these sections.
- The research gap is identified but could be made sharper. Explicitly state: “No prior studies quantitatively evaluated national park policy texts in China using NLP and grounded theory.”
- Some references are outdated (e.g., Curry 2009 [2]). Add more recent global studies (post-2020) on national park governance and policy analysis.
- Clearly state the novelty: that this is the first quantitative evaluation of Chinese national park policy texts using NLP and grounded theory.

3. **Literature Review**

- The review is comprehensive but reads as a list of studies. Please strengthen thematic synthesis and explicitly connect prior work to your methodological choices.
- The review reads more like a list of studies rather than a critical synthesis. Try to connect them thematically (e.g., “Most previous work used the PMC-Index, which has limitations…”).
- Include more explicit justification of how your mixed-method approach addresses existing methodological shortcomings.
- Some citations are repeated (e.g., Yochim & Lowry 2016 appears twice in slightly different contexts).
- Include more recent international studies (post-2020) to enhance relevance.

4. **Materials and Methods:**

- Provide more detail on data collection: search terms, inclusion/exclusion criteria, and sources.
- The methods section is quite technical but lacks reproducibility details. For example: Provide exact search terms and inclusion/exclusion criteria for policy texts; Clarify whether coding reliability (e.g., Cohen’s kappa) was assessed; Specify the Python packages used for NLP and weighting.
- Specify the software and Python packages used.
- Figures (e.g., Fig 1 and Fig 2) need clearer captions and higher resolution.
- The equations (e.g., policy response evaluation model) should be formatted more consistently.

5. **Results:**

- Some results overlap with interpretation. Consider moving interpretive sentences to the discussion section.
- Tables (e.g., Table 3 and 4) are very dense; consider summarizing in text or visualizing more results with figures.
- Ensure standard deviations and other statistics are adequately explained (why they matter for heterogeneity).

6. **Discussion, Implications, and Findings:**

- The discussion is somewhat repetitive of the results. More emphasis should be placed on the novel insights for international audiences (e.g., how China’s centralized-local hybrid system contributes to global policy lessons).
- Expand the comparative angle: how might lessons from China apply to other developing countries establishing national parks?
- Consider integrating critical reflection on potential biases in policy text analysis (e.g., policies may not always reflect actual implementation).

7. **Limitations, Future Research Directions:**

- The conclusion could be more concise and highlight three key takeaways for readers.
- Limitations section should mention potential bias due to reliance on publicly available policy documents (some internal policies may be inaccessible).
- Future research suggestions could emphasize comparative international studies, not just extensions in China.

8. **References:**

- Some DOIs appear duplicated (e.g., [2] lists two DOIs).
- Consider pruning less relevant references to tighten the review.
- Include more recent references (2020–2024), verify that all in-text citations appear in the reference list and vice versa.
