# Supplementary material for: Research on the management of the system construction of National parks with China characteristics: Evidence from policy texts
Source: PLoS One. 2026 Mar 2;21(3):e0340874. doi: 10.1371/journal.pone.0340874 (PMC12952615; doi:10.1371/journal.pone.0340874)
Supplement: Attachment — Submitted filename: 1112+Responses to reviewers-PONE-D-24-46176.docx [file pone.0340874.s008.docx]

October 16, 2025

**RE: PONE-D-24-46176**

Title: Research on the Management of the System Construction of National Parks with China Characteristics: Evidence from Policy Texts

Dear Reviewers:

We would like to thank you for the opportunity to resubmit a revised copy of this manuscript. We greatly appreciate your valuable comments and suggestions. As per your requests, we have carried out the revisions that you suggested and revised the manuscript accordingly.

Please find a point-by-point response to your concerns uploaded alongside this cover letter. Additionally, it should be noted that we have had the revised manuscript reviewed by an experienced and native English-speaking editor for another round of professional editing. We assure you that the manuscript has been checked for correct use of grammar and standard technical terms and edited to a level suitable for reporting research in the *PLOS One*. We hope you find our responses satisfactory and that the revised manuscript meets your standard and is now acceptable for publication in the *PLOS One*. Thank you.

Once again, we acknowledge your feedback very much, which is valuable in improving the quality of our manuscript.

Sincerely yours,

The Authors

Response to Reviewer ’s Comments

Manuscript: PONE-D-24-46176

Title: Research on the Management of the System Construction of National Parks with China Characteristics: Evidence from Policy Texts

The authors would like to thank the reviewer for xyour time and comments. Your feedback is very important. We have carefully addressed each of your suggestions. The corresponding answers for each comment from Reviewer are highlighted in RED below and in the revised manuscript. We hope that these clarifications and major additions will now convince you.

---------------------------------------------------------------------------------------------------------------------

| **Reviewer’s Comments** | **Responses/Revisions Made** |
| --- | --- |
| 1. Abstract:  • The abstract should include a sentence on the significance of this study for broader international audiences (why non-China readers should care). | The authors agreed with the reviewer’s comment and appreciated your calling our attention to this constructive suggestion. Follow your suggestions, we have expanded the description of this study's national significance:  We have revised the sentence “A mixed-method approach, combining natural language processing and grounded theory, is utilized to develop a quantitative evaluation framework for analyzing national park policy texts”. And now it reads “*Quantitative evaluation of China’s national park policy texts is an important window to understand its national park governance logic, top-level design of ecological protection, and future practice.”(***Lines 12-14** on page 1:*)*  We have revised the sentence *“*This study contributes to the global understanding of national park policy evaluation, offering insights that can inform both Chinese and international contexts”. And now they read *“Its findings offer valuable inspiration for developing countries to establish national parks protection systems and provide direct reference for global biodiversity governance. And it will also play a key role in achieving the action-oriented global goals of Kunming-Montreal Global Biodiversity Framework*.*” (***Lines 23-26** on page 1*)* |
| 1. Abstract:  • Some sentences in the abstract are long and could be shortened for clarity. | We are appreciative of the reviewer’s comments. Follow your suggestions, we have shortened certain sentences to enhance clarity,such as:  We have revised these sentences“This study is based on an analysis of 77 national park policies in China, focusing on three key areas: the central government's top-level design of national parks, local government responses, and the heterogeneity of these responses. The findings reveal that:”. And now they read “*This study focuses on three key areas: the central government's top-level design of national parks, local government responses, and the heterogeneity of these responses.* *By analyzing 77 national park policies in China, the following conclusions are drawn:”* *(***Lines 14-17** on page 1*)* |
| 2. Introduction:  • The introduction is lengthy and contains excessive descriptive detail (e.g., average park sizes). Consider condensing these sections. | We are appreciative of the reviewer’s comments. Following your suggestions, the description was deleted from the revised manuscript.  We have removed the sentence “To date, the average size of the first five national parks in China is approximately 46,000 square kilometers, significantly larger than the average size of the 62 U.S. national parks, which is less than 3,400 square kilometers.” |
| 2. Introduction:  • The research gap is identified but could be made sharper. Explicitly state: “No prior studies quantitatively evaluated national park policy texts in China using NLP and grounded theory.” | We are appreciative of the reviewer’s comments. Following your suggestions, we explicitly state that “And *no prior studies quantitatively evaluated national park policy texts in China using NLP and grounded theory*” (**Lines 80-82** on page 3) in the manuscript. |
| 2. Introduction:  • Some references are outdated (e.g., Curry 2009 [2]). Add more recent global studies (post-2020) on national park governance and policy analysis. | We are appreciative of the reviewer’s comments. Following your suggestions, we have removed outdated references and incorporated additional recent global research on national park governance and policy analysis, such as:  **Lines 68-72** on page 3:*Zhu et al. (2024) use the multiple streams framework as a theoretical and through the lens of policy entrepreneurs, to furnish a renewed look at the policymaking process of conservation policy in China. Chai et al. (2025) proposes a triadic “Natural-Social-Digital” framework for assessing China’s national park policy effects.* |
| 2. Introduction:  • Clearly state the novelty: that this is the first quantitative evaluation of Chinese national park policy texts using NLP and grounded theory. | We are appreciative of the reviewer’s comments. Following your suggestions, we clear state that “*it is the first quantitative evaluation of Chinese national park policy texts using NLP and grounded theory*” (**Lines 92-93** on page 4) . |
| 3. Literature Review  • The review is comprehensive but reads as a list of studies. Please strengthen thematic synthesis and explicitly connect prior work to your methodological choices. | To address your concerns, we have collated existing research findings and synthesized them by topic. For example, we conducted a thematic review of the research results on national park policy, focusing on three aspects: national park policy tools, challenges, and goals. This has enabled us to clearly delineate the limitations of these methods and elucidate the innovative aspects of this research.  *When we sorted out national park policy tools, we found that the initial focus was on individual policies such as law or economics. This was followed by a gradual transition to a combination of policy instruments. As Kubo et al. (2019) [22] observed, strategically combining policy instruments is essential for enhancing national park policy effectiveness. However, existing research has not established a systematic framework to evaluate the interaction of different policy instruments in complex governance situations. Especially for a country as vast as China, it remains unclear how to optimize the tool mix to coordinate central goals and local diversity in national park development. This theoretical gap prompts this article to adopt a research method that combines NLP and grounded theory.(* **Lines 130-137** on page 5*)*  The reference section contains newly cited sources. |
| 3. Literature Review  • The review reads more like a list of studies rather than a critical synthesis. Try to connect them thematically (e.g., “Most previous work used the PMC-Index, which has limitations…”). | To address your concerns, We try to synthesize policy text analysis methodologies through two principal approaches: single-policy evaluation techniques and the PMC Index. At the same time, we critically synthesize existing research findings, identifying shortcomings in prior studies and highlighting the innovative contributions of this paper..  *In collating existing research on the PMC Index, we observed that most previous studies use PMC-Index, which has significant limitations in setting policy variables and weights. First, the evaluation results are highly dependent on the preset variable system and may not capture the unique regional concepts and governance logic in the policy. While, we used NLP to analyze 77 national park policies. At this stage, the aim is to naturally identify core policy clusters from large-scale text data, avoid preset framework bias, and ensure that research conclusions are rooted in the original texts.. (***Lines 195-201**on page7*)*  The reference section contains newly cited sources. |
| 3. Literature Review  • Include more explicit justification of how your mixed-method approach addresses existing methodological shortcomings. | To address your concerns, we include more explicit justification.  **Lines 221-226** on page 7-8: *In the analysis process (see below for specific steps), macro-level NLP findings provide direction for micro-level grounded theory research, and the coding results of grounded theory can feed back to the NLP model for verification and optimization. For example, word vector models can be retrained based on new recognition concepts. This iterative process significantly improves the reliability and interpretability of research results [51]*. |
| 3. Literature Review  • Some citations are repeated (e.g., Yochim & Lowry 2016 appears twice in slightly different contexts). | To address your concerns, we have revised and adjusted the recurring quotations. |
| 3. Literature Review  • Include more recent international studies (post-2020) to enhance relevance. | To address your concerns, as amended above, we have included more recent international studies to enhance relevance. For example:  *Regarding economic instruments, studies have investigated fee policies [19], eco-compensation [20] and tourism promotion [21] as mechanisms connecting national park ecological value with public awareness.(* **Lines 128-139** on page 5*)*  The reference section contains newly cited sources. |
| 4. Materials and Methods:  • Provide more detail on data collection: search terms, inclusion/exclusion criteria, and sources. | We value your input. Following your instructions, we provide more detail on data collection:  We have revised the sentence “A search using the term “national park” yielded 424 policy texts, which were reviewed to exclude low-relevance and redundant documents, 77 valid texts to be retained for the corpus.” And now they read “*A search using the term ‘national park’ yielded 424 policy texts. Filtered to exclude invalid text, text missing the issuing authority, document number or publication date. At the same time, meeting notices, duplicate policies, approval letters, and multi-batch catalog policies are eliminated. Additionally, less relevant documents were eliminated, such as bank announcements regarding specific national park coins and industry regulations covering the sale of such coins. Finally, 77 valid texts were retained in the corpus*”. (**Lines 224-230** on page 8) |
| 4. Materials and Methods:  • The methods section is quite technical but lacks reproducibility details. For example: Provide exact search terms and inclusion/exclusion criteria for policy texts; Clarify whether coding reliability (e.g., Cohen’s kappa) was assessed; Specify the Python packages used for NLP and weighting. | We value your input. Following your instructions, in addition to the aforementioned modifications, we also clearify whether coding reliability was assessed. **Lines 265-270** on page 9-10: “*The research team first conducted a pilot coding exercise to establish an overall coding framework. Subsequently, two research assistants independently coded 77 documents based on this framework. Coding reliability was tested using Cohen's kappa coefficient for consistency. After testing, it was found that the kappa coefficient of the two researchers coding the national park policy text was 0.812(>0.8). It can be seen that the coding results of this time are reliable.*” *Table 1 Classification standard of Cohen's kappa coefficient* Cohen's kappa coefficient \| <0.2 \| 0.21~0.40 \| 0.41~0.60 \| 0.61~0.80 \| 0.81~1.00; Level of consistency \| Poor \| Weak \| Moderate \| High \| Excellent |
| 4. Materials and Methods:  • Specify the software and Python packages used. | We value your input. Following your instructions, we specify the packages in Python sofeware and Python, for example jieba.posseg for Part-of-speech tagging, jieba.analyse for Keyword Extraction, SnowNLP for natural language processing. (**Lines 2262-263** on page 9) |
| 4. Materials and Methods:  • Figures (e.g., Fig 1 and Fig 2) need clearer captions and higher resolution. | We value your input. Following your instructions, we have recalibrated all the figures. For example:    Fig 2. Research process and main steps. |
| 4. Materials and Methods:  • The equations (e.g., policy response evaluation model) should be formatted more consistently | We value your input. Following your instructions, we have adjusted the format of the equations:  $\text{Y}_{\text{k}}\text{=}\sum_{\text{j=1}}^{\text{n}} \text{w}_{\text{j}}\text{X}_{\text{j}}$ |
| 5. Results:  • Some results overlap with interpretation. Consider moving interpretive sentences to the discussion section. | Taking your advice into account, we have revised the Discussion section. This revision involved removing explanatory statements, such as the sentence “In the strategic planning of national parks, elevated average scores were observed in areas such as integrated management, environmental oversight, organizational leadership, and evaluation monitoring” Furthermore, the Discussion has been reframed to incorporate an international comparative perspective, a critical reflection on the limitations of text-based analysis, and a deeper interpretation of China-specific findings, thereby significantly enhancing the study's rigour and relevance. |
| 5. Results:  • Tables (e.g., Table 3 and 4) are very dense; consider summarizing in text or visualizing more results with figures. | Taking your advice into account, we have visualised Tables 3 and 4 in the form of charts and tables.  **Fig 3.** The comparison of average scores for primary variables between the top-level design and local response. |
| 5. Results:  • Ensure standard deviations and other statistics are adequately explained (why they matter for heterogeneity). | Taking your advice into account, we have provided a thorough explanation of the standard deviation.  **Lines 353-357** on page 12-13: “*As a core indicator in statistics, standard deviation is a statistical measure that gauges the dispersion of values in a series relative to the mean, serving as the root of the variance [58]. It intuitively reflects the variability and volatility of the data set: higher values indicate more significant data fluctuations, and lower values mean weaker volatility.*” |
| 6. Discussion, Implications, and Findings:  • The discussion is somewhat repetitive of the results. More emphasis should be placed on the novel insights for international audiences (e.g., how China’s centralized-local hybrid system contributes to global policy lessons). | We agree with this comment. And we have revised the discussion and added the novel insights for international audiences.  We have revised the sentence “Line 447-452 on page 16”. And now they read “*Global discourse on protected area governance often presents a binary opposition between the perceived effectiveness of centralized control and the flexibility of decentralization [59]. Our analysis of China's national park policy documents, however, reveals a viable hybrid model. This model integrates mandatory ecological protection "red lines" set by the central government—which serve as non-negotiable baselines—with the flexibility for local governments to explore diverse implementation pathways above these thresholds, such as concession management and ecological compensation mechanisms. This "hard bottom line with flexible space" framework facilitates central-local collaborative governance, ensuring national strategic unity while stimulating local initiative, thereby offering valuable insights for global biodiversity conservation and ecological governance.*” (**Lines 466-476** on page 16-17) |
| 6. Discussion, Implications, and Findings:  • Expand the comparative angle: how might lessons from China apply to other developing countries establishing national parks? | We agree with this comment. And we have added the comparative angle.  We have revised the sentence “Line 453-463 on page 16”. And now they read “*Balancing nature conservation with local socioeconomic development presents a global challenge, particularly in developing countries [60]. As the world's largest developing country, China's national park system offers relevant insights. Initiatives such as concession management, ecological compensation, and the "one household, one industry" program have effectively fostered local community participation. By leveraging their regional management strengths, local governments can further establish a collaborative governance framework that integrates administrative agencies, non-profit organizations, and community residents [61]. This approach helps optimize stakeholder participation and generate synergistic effects for conservation and development.”* (**Lines 477-486** on page 17) |
| 6. Discussion, Implications, and Findings:  • Consider integrating critical reflection on potential biases in policy text analysis (e.g., policies may not always reflect actual implementation). | We agree with this comment. And we have considered integrating critical reflection.  We have revised the sentence “Line 464-474 on page 16-17”. And now they read “*Although the national park policy framework provides a clear blueprint for central-local coordination, several implementation challenges warrant in-depth examination. It is important to note that our analysis, based on policy documents, inherently carries the limitation that written policies may not fully capture on-the-ground realities. First, a tension exists between policy uniformity and implementation diversity. While master plans employ similar structural frameworks, regional variations in land tenure, community composition, and economic development necessitate adaptive local practices, potentially leading to divergent outcomes from uniform policies. This gap between textual prescription and practical adaptation underscores a key limitation of purely document-based analysis. Second, entrenched sectoral path dependence may undermine the policy's "unified management" goal. Within the existing administrative structure, high costs of cross-departmental coordination can result in goal deviation or implementation inefficiencies, challenges that policy texts themselves may acknowledge but cannot fully resolve in practice. Finally, the effectiveness of community engagement requires continuous monitoring. The long-term sustainability of "shared benefits" outlined in policy texts depends on whether communities secure substantial and equitable economic returns and achieve meaningful representation through ecotourism and concession schemes. This highlights the critical distinction between the normative aspirations embedded in policy and their tangible realization.*” (**Lines 487-505** on page 17-18) |
| 7. Limitations, Future Research Directions:  • The conclusion could be more concise and highlight three key takeaways for readers. | We agree with this comment. The conclusions section highlights three key points.  **Lines 552-557** on page 20: “*Our results revealed that: The central government's planning priorities for the national park system focus on ecological conservation and sustainable livelihoods for indigenous communities; (2) Significant variations exist in the implementation of national park policies by local governments; (3) The five inaugural national parks exhibit marked differences in conservation objectives, integrated management practices, spatial planning, and industrial development trajectories*”. |
| 7. Limitations, Future Research Directions:  • Limitations section should mention potential bias due to reliance on publicly available policy documents (some internal policies may be inaccessible). | Dear respected reviewer: we have made adjustments to the restricted section.  **Lines 562-565** on page 20: “*Due to time constraints and limited access to some government websites, this study was unable to obtain all national park-related policy documents in China. Therefore, the study results may be subject to potential bias.*”. |
| 7. Limitations, Future Research Directions:  • Future research suggestions could emphasize comparative international studies, not just extensions in China. | Dear respected reviewer: we have revised our recommendations for future research accordingly.  **Lines 567-571** on page 20: “*Future efforts should further strengthen comparative research, particularly comparative research on national park policy texts between countries with different political systems or different levels of development, and further explore their differences and underlying causes, thereby providing successful experience for global ecological governance*”. |
| 8. References:  • Some DOIs appear duplicated (e.g., [2] lists two DOIs). | Dear reviewer, we appreciated your time in making this comment. We have removed instances of duplicate DOIs（e.g., [2]） and adjusted the corresponding duplicate references. |
| 8. References:  • Consider pruning less relevant references to tighten the review. | Dear reviewer, we appreciated your time in making this comment. We have compiled a list of less pertinent references, such as those pertaining to policy texts, which have been excluded from the relevant research section.  We have removed the reviem, such as:  Alipour, M., Hafezi, R., Ervural, B., Kaviani, M. A., Kabak, Ö. (2018). Long-term policy evaluation: Application of a new robust decision framework for Iran's energy exports security. *Energy* ,*157*, 914-931. <https://doi.org/10.1016/j.energy.2018.05.176>  Guo, Y., Fu, B., Xu, P., Wang, Y., Liu, X. (2021). Mapping regional differences in payment for ecosystem service policies to inform integrated management: Case study of the Yangtze River Economic Belt. Journal of Environmental Management, 278: 11396. <https://doi.org/10.1016/j.jenvman.2020.111396>  Huang, B., Choi, Y., Chng, S., Neo, H. (2023) Examining Policy Strategies for Electrifying Transportation in ASEAN: A STEELUP Framework Evaluation. *Sustainability* , *15* (22) *:*15708. <https://doi.org/10.3390/su152215708>.  Huang, X., Zhu, R., Wu, X., Ge, P. (2023). Assessing the role and driving mechanisms of the green financial reform on urban energy consumption and pollution emissions: a policy evaluation from the generalized synthetic control method. *Environmental Science and Pollution Research*, *30* (56), 119095-119116. https://doi.org/10.1007/s11356-023-30482-z  and so on. |
| 8. References:  • Include more recent references (2020–2024), verify that all in-text citations appear in the reference list and vice versa. | Dear reviewer, we appreciated your time in making this comment.We have incorporated recent references (2020–2024) into the paper and verified all citations to ensure they appear in the bibliography.  We added the current referenct, such as:  [Brumatti](https://www.researchgate.net/profile/Paula-Brumatti-2?_tp=eyJjb250ZXh0Ijp7ImZpcnN0UGFnZSI6InB1YmxpY2F0aW9uIiwicGFnZSI6InB1YmxpY2F0aW9uIn19) PN, Rozendo C. National Parks, tourism and governance: Reflections on tourism concessions in Brazil [J]. Revista Brasileira de Pesquisa em Turismo, 2021, 15(3):2119. <https://doi.org/10.7784/rbtur.v15i3.2119>  Song L, Xue Y, Jing Y, & Zhang J. Visitor’s Willingness to Pay for National Park Entrance Fees in China: Evidence from a Contingent Valuation Method [J]. International Journal of Environmental Research and Public Health, 2021, 18(24): 13410. <https://doi.org/10.3390/ijerph182413410>  Flton E, Mels T. Historical boundary struggles in the construction of the non-human world: Nature conservation and tourism in Swedish national parks [J].Journal of Historical Geography, 2024, 86:70-81. <https://doi.org/10.1016/j.jhg.2024.06.006>  Shokirov Q, Backhaus N, Bartmess J. Community perceptions of co-managing Tajik National Park [J]. Central Asian Survey, 2021(2):1-18. <https://doi.org/10.1080/02634937.2021.1942787>  Järv H, Ward RD, Raet J, Sepp K. Socio-Economic Effects of National Park Governance and Management: Lessons from Post-Socialist Era Estonia [J]. Land, 2021, 10(11): 1257. <https://doi.org/10.3390/land10111257>  and so on.  Once again, we very much valued the reviewer’s insights and are grateful to the reviewer for bringing this comment to our attention. Thank you. |

Average score

| Primary variables | Central average score | Local average score |
| --- | --- | --- |
| Policy basis | 10.4435 | 12.2993 |
| Policy objectives | 12.5452 | 50.2658 |
| Policy measures | 138.94 | 383.671 |
| Policy receptors | 9.70626 | 31.1254 |
| Policy areas | 38.6226 | 194.287 |
| Policy functions | 23.5724 | 60.2911 |
| Policy guarantee | 21.767 | 51.0856 |

Again, to Reviewer , we thank you very much for your kind inputs. We have also learned a lot from your thoughtful comments, which have helped improve this study’s contents.

- The Author
